# Supplementary material for: The salmon louse genome may be much larger than sequencing suggests
Source: Sci Rep. 2022 Apr 22;12:6616. doi: 10.1038/s41598-022-10585-2 (PMC9033869; doi:10.1038/s41598-022-10585-2)
Supplement: Supplementary file 1 — Supplementary Information. [file 41598_2022_10585_MOESM1_ESM.pdf]

The salmon louse genome may be much larger than sequencing suggests

Grace A. Wyngaard, Rasmus Skern-Mauritzen, Ketil Malde, Rachel Prendergast, Stefano Peruzzi

Supplementary Information

Materials and methods

Flow cytometry (FCM) and Feulgen image analysis densitometry (FIAD) methodologies

FCM uses light scatter of individual cells passed through a laser beam in a suspension, producing peaks of counts that can be interpreted as various ploidy levels. FIAD relies on the Feulgen reaction to quantify the intercalation of stain among nucleotides, and thus light passing through the nucleus. Occasionally a diversity of staining intensities, visible as densely staining nuclei attributable to DNA compaction and very diffuse appearing nuclei, may underestimate or overestimate, respectively, DNA content. Thus, FIAD sometimes involves more subjectivity on part of the investigator; however, it allows specific tissues to be easily targeted within a single specimen and may be the method of choice when the number of cells available to measure is small. Jeffrey (2015) reported that in crustaceans the leg muscle was optimal for FCM while gill tissue was optimal for FIAD. Copepods lack gills, but a few nuclei in the spine of an appendage of the Maine population were especially well prepared histologically and served as a comparison between wild caught

Maine and Tromsø populations as well as between wild caught and laboratory strains. The standard methods used to squash and prepare isolated nuclei for FIAD in studies of free-living copepods (e.g. Rasch & Wyngaard, 2006) did not sufficiently separate the cellular layers from the thick carapace of parasitic copepods. Freeze-cracking solved this problem in squash preparations of *L. salmonis salmonis* and low coefficients of variation within an individual were obtained. Freeze-cracking may prove helpful in other tissues and species that do not readily lend themselves to standard FIAD protocols.

Nuclear DNA content analysis by flow-cytometry (FCM)

Field and laboratory populations. Specimens of *L. salmonis salmonis* were obtained from several sources: (1) Wild adult males and females were collected from naturally infected farmed Atlantic salmon held at the sea cage facilities of the Aquaculture Research Station in Tromsø (Norway, 69°N, 19°E) (FCM Run 5). (2) An outbred laboratory strain, *Ls* Gulen, was derived from adults collected in Gulen, Sogn og Fjordane, Norway (60°N, 58°E) in June 2006 and at reared at the Salmon Louse Research Centre in Bergen (FCM Run 4). (3) A hybrid laboratory strain was established by crossing adults from the *Ls* Gulen strain with a partially outbred strain, *Ls* Oslofjord, which was established from collections in Oslofjord, Norway (59°N, 10°E) in June 2006. This hybrid strain, referred to as the Ls Tromsø strain in the present study, was reared at the Aquaculture Research Station in Tromsø for ca. 25 generations (FCM runs 1, 2 and 3). Additional details about the laboratory strains are provided in Hamre *et al.* (2009).

Collection of samples and tissue preparation. Newly hatched nauplii were obtained from gravid females from the Ls Tromsø strain (FCM Runs 1 and 2) bearing well-developed egg strings and kept in individual 200 ml beakers with filtered seawater in a temperature-controlled room at 5°C. Approximately n = 50 nauplii/female were collected using a 100 µm mesh filter

(Sysmex Partec GmbH, Germany), quickly rinsed with cold distilled water and transferred to a 1.5 ml Eppendorf tube containing 0.5 ml cold citrate buffer composed of 5% dimethyl sulfoxide (DMSO), 250 mM sucrose, and 40 mM sodium citrate at pH 7.6. This solution allows permeabilization and preservation of nuclear membranes (Vindeløv *et al.*, 1983). After addition of 0.5 ml fresh citrate buffer, the nauplii were then crushed on ice using an Eppendorf micropestle, filtered through a 20 μm mesh filter (Sysmex Partec GmbH, Germany) and the samples were then stored at -80°C until use (approximate storage time of 1 week).

Sperm and eggs of the Ls Gulen strain (FCM Run 4) were collected from the testes and the genital segment prior to fertilization, respectively, using a scalpel and glass pipette under a stereoscopic microscope and directly transferred to an Eppendorf tube containing 1 ml citrate buffer. Samples were held briefly on ice prior to flow cytometry analysis.

Adult tissues were obtained by excising small pieces of cuticle and underlying tissues from the cephalic region of *L. salmonis salmonis* (FCM Runs 3 and 5) with a scalpel and viewed using a stereoscopic microscope (Supplementary Fig. 1). This procedure allowed collecting sufficient number of somatic tissue (cells) for flow cytometry analysis while avoiding gonadal tissue. The somatic tissues were transferred to a 1.5 ml Eppendorf tube containing 0.5 ml cold citrate buffer, crushed, and treated in the same way as the newly hatched nauplii (see above).

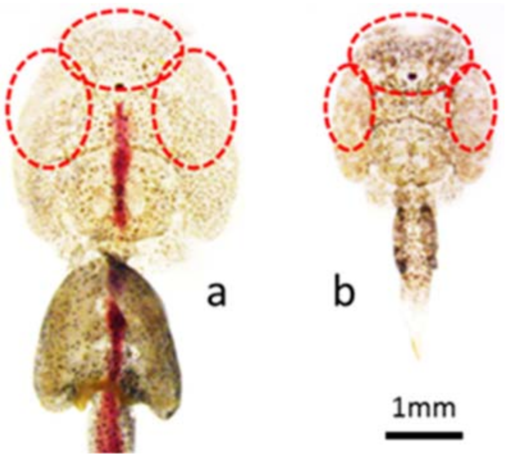

Supplementary Figure 1. Adult female (a) and male (b) *L. salmonis salmonis* collected from farmed Atlantic salmon. Red dotted lines show the cephalothorax regions from which cuticular and subcuticular tissues were sampled.

Internal standards consisted of blood cells of a male human and chicken. A 1 ml sample of fresh male human (*Homo sapiens*) mono-nucleated white blood cells (MNCs, total operator count 82.7 x 10^6/ml) was obtained by centrifugation of peripheral blood over a density medium following in-house methods (Department of Immunology, Transfusion Medicine, University Hospital of North Norway, Tromsø), and then was diluted 1:1 with citrate buffer and stored in 200 µl aliquots at -80°C until use. Chicken blood was obtained from the brachial vein of a rooster (*Gallus domesticus*) using a BD Vacutainer®EDTA equipped with a 23-gauge needle. Whole blood was centrifuged (2000 rpm, 3 min), plasma removed and packed cells re-suspended back to the original blood volume with physiological saline (BioUltra, Merck KGaA, Darmstadt, Germany). The blood sample (chicken erythrocyte nuclei, CEN) was then mixed with an equal volume of the citrate buffer and stored in 200 µl aliquots at -80°C until analysis.

Samples from target species (salmon lice) and reference species (male human and chicken) were first mixed in equal proportions and then assayed simultaneously, as described by Tiersch *et al.* (1989). Aliquots of target and reference cells were suspended in 1ml of Propidium Iodide (PI) staining buffer (Tiersch *et al.*, 1989) containing 50 mg/l of PI (cat. no. P-4170, Sigma Aldrich) and 50 mg/l of RNase A (Roche Diagnostics GmbH, Germany), mixed and filtered through a 30 µm nylon mesh. Prior to analysis, samples were kept at +4 ◦C in the dark for 45 min to ensure thorough staining of nuclei.

The mean DNA content of 5000 – 10,000 cells per sample was measured with a CyFlow®Ploidy Analyser (Sysmex Partec GmbH, Germany) equipped with a green laser (Nd-YAG laser 532 nm emission wavelength, 30 mW). Data are presented in arbitrary units expressed as fluorescence (FL) channel numbers along a linear scale. Nuclear DNA contents of target species were estimated in relation to an assigned 2*C* value of 7.00 pg DNA/nucleus for male human leukocytes and 2.50 pg DNA/nucleus for chicken erythrocytes (Gregory, 2021) according to the formula:

Target species nuclear DNA content (pg) = (Mean FL value of the sample × Standard 2*C* DNA content) × (Mean FL value of the standard)$^{-1}$

Each day, prior to data acquisition, a standard procedure was used to check the optical alignment and instrument linearity by use of fixed, PI-labelled trout erythrocytes (DNA Control PI, Sysmex Partec GmbH, Germany) providing peaks for single nuclei, doublets, and a few larger aggregates. The flow cytometer's sensitivity and range were analysed using reference fluorescent beads (Calibration Beads green Concentrate, Sysmex Partec GmbH, Germany) following procedures recommended by the manufacturer. Windows™ XP software for data acquisition and Cyflogic v.1.2.1 software (Perrtu Terho & ©CyFlo Ltd) for peak analysis were used. The mean nuclear

DNA contents are reported as 2*C* values in picograms (pg) and converted to gigabase (Gb) pairs (1 pg DNA = 0.978Gb) (Doležel *et al.*, 2003).

Feulgen image analysis densitometry

Field and laboratory populations. Tissues for genome size measurement were obtained from each of three adults (females) of the of the Ls1a laboratory strain, whose ovaries served as the source material of DNA used in the nanopore DNA sequencing and six adults (three males and three females) of *L. salmonis salmonis* from *Ls* Gulen laboratory strain used in the FCM studies (see above). A single adult female was collected from the wild in Copscook Bay, Maine in 2018 and used in comparison with wild *L. salmonis salmonis* collected from Tromsø, Norway. Norway specimens were immediately preserved in undenatured >99% alcohol; Maine specimen, in undenatured >95% alcohol.

Tissue preparation and freeze-flipping. Due to the tough and thickened cuticle of some parasitic copepods and the propensity for cells to adhere to the cuticle, freeze-cracking was added to the squashing procedure commonly used for isolating the nuclei of free-living copepods. The typical method for preparing tissues of free-living copepods such that isolated nuclei are obtained (according to Clower *et al.*, 2016) does not yield isolated and high-quality spreads of nuclei of parasitic copepods. To remedy this, portions of the copepod body were subjected to freeze-cracking using a procedure modified after Duerr (2013). Freeze-cracking separates the cuticle from its underlying tissues by compressing the organism between a glass slide and a siliconized coverslip, freezing on a liquid nitrogen table, and then flipping the coverslip to expose the tissues to subsequent chemical treatments. We believe this is the first publication describing estimation of

genome size using freeze-cracking and Feulgen image analysis densitometry and so have presented a detailed description of tissue preparation. The single female specimen from Maine included in the present study was not subjected to freeze-cracking and as a result, only nuclei in an appendage were suitable for measurement.

Small pieces of the cephalic region of each *L. salmonis salmonis* were placed in a glass depression slide with undenatured 190 proof ethanol and sliced with tungsten probes to obtain a few smaller, more flattened pieces of carapace and tissue. Tissues were transferred to a glass slide using flexible insect forceps, overlaid with a siliconized coverslip (treated with Sigmacote), and squashed with a mechanical press. A portion of the coverslip was allowed to hang off the slide. Freezing was performed on a liquid nitrogen table for about five minutes, and was immediately followed by flipping the overhanging edge of the coverslip with blunt-nosed thumb forceps. The carapace and tissues were then transferred to a depression slide containing 3:1 ethanol:acetic acid for approximately 2 min, and then immediately transferred to a depression slide with a drop of 45% acetic acid (to minimize carry-over of alcohol) for approximately 3 min, followed by transfer to a subbed slide and quickly teased apart further with tungsten probes.. Treatment in acetic acid for more than three to four minutes typically resulted in wispy, degraded nuclei which did not provide accurate measurements of DNA content in previous squashing and training trials. Tissues were then squashed under a siliconized coverslip, with the goal of obtaining spreads one cell layer thick. The tissues were treated to a second freeze-flip, and the slides were oriented at an angle with tissues facing downward and allowed to air dry. After drying completely, the slides were rinsed in two washes of 200 proof ethanol to remove acetic acid residues. Allowing the slides to dry completely before the ethanol washes was imperative in order for tissues to adhere to subbed slides. After each ETOH wash (2 – 3 min each), the slides were again air dried, and stored in the dark

until staining. This series of steps prior to Feulgen staining that were modified from Clower *et al*. (2016) yielded improved quality of tissue preparations with respect to the dispersion and number of nuclei suitable for measurement. Prior to staining, all slides were treated to a second post-fixation in methanol:formalin:acetic acid (85:10:5) (MFA) solution for 10 minutes followed by rinsing in tap water for 10 minutes.

Staining using the Feulgen reaction. Feulgen image analysis densitometry was used to quantify the amounts of DNA in nuclei of diploid somatic cells. This method allows one to identify the particular nuclei being measured and sometimes their location, such as an appendage, within the body. It is well suited for tissues of microscopic organisms and does not rely on availability of very large numbers of cells.

Standards. Reference standards for conversion of IOD units to picograms included mutant white eyed female *Drosophila melanogaster* (0.40 pg DNA per nucleus), erythrocytes of hen *Gallus domesticus* (2.5 pg DNA per nucleus) and trout *Oncorhyncbus mykinss* (5.2 pg DNA per nucleus), and human mono-nucleated cells of male human *Homo sapiens* (7.0 pg DNA per nucleus). Blood for the standards were obtained from the brachial vein of a chicken (*Gallus domesticus*) and the heart of a trout (*Oncorhynchus mykiss*) equipped with a 23-gauge needle to which heparin was added. Blood smears were made by streaking a drop of blood onto acid washed glass slides. These standard preparations were made on a single date in the 1990's, stored in the dark at room temperature, and used in all subsequent staining procedures in the Wyngaard lab. The standard *D. melanogaster* was freshly prepared by narcotizing the entire body with CO_2, removing the heads with forceps, and transferring the heads to a 1% sodium citrate in phosphate-buffered saline (PBS), followed by permeabilization in 1% Triton solution in PBS solution 30 seconds. The tissues of the head were teased apart and squashed as described above for copepods. A whole blood

sample from a human male was treated with Histopaque and then centrifuged using a Thermo Scientific Cytospin 4 to separate and apply a thin layers of cells on slides.

Feulgen reaction – All slides were stained with Schiff reagent according to Hardie *et al.* (2002) and Clower *et al.* (2016), except that the slide preparations of the Ls1a specimens were treated to a second post-fixation in 85:10:5 methanol:formalin:acid solution. Hydrolysis of tissues was conducted at 23°C for 20 minutes, a temperature and time that was optimized for copepod tissues. Stained slides were air-dried and stored for up to one month prior to measurement of genome size.

Densitometry. Nuclei were viewed and measured using a Zeiss Axioscope A1 equipped with a 63X oil objective (1.515 refraction index, Cargille Laboratories, Inc.) and a Qimaging Bioquant PVI CCD camera. Scanning microdensitometric software (Bioquant Image Analysis; Bioquant Life Sciences 2018 program) was used to determine the integrated optical densities (IODs) of the nuclear DNA contents of individual somatic nuclei taken from the cephalic region of each *L. salmonis* specimen. *L. salmonis* specimens and standards from *D. melanogaster*, chicken and trout were measured using the green channel that possesses a peak absorbance approximating 540 nm. Human blood standards were measured using a custom filter (Chroma Technology Corp.) with a peak absorbance of 605 nm and a bandpass of 50 nm. This use of a longer wavelength to measure relative large genomes was necessitated by the CCD camera used with the Bioquant software and is comparable to measuring the nuclei “off peak” as described in Rasch (1985). We selected for measurement only nuclei that possessed a granular and slightly diffuse appearance and lacked visible pink background; these nuclei were found mostly at the perimeter or outside the carapace (Fig. 2C,D). Nuclei with relatively small areas and dense staining indicating DNA compaction (Fig. 2E) or very diffuse and large areas (Fig. 2F) are less likely to yield accurate measurements. These subjective choices of nuclei to measure are based on experience in measuring thousands of

nuclei from free-living copepods (G.A. Wyngaard, pers. observation). Typically, measurements for a particular specimen were obtained from several views of the slide. The lower and upper 5% of the range of IOD values were excluded when calculating the mean IOD value. Coefficients of variation within the populations of nuclei in the *Ls1a* samples and the four standards varied from 3.9 – 10.2% and 3.5 – 3.9%, respectively. The calibration curve computed for these four standards in the staining batch containing the *Ls*1a strain yielded an $R^2 = 0.997$ (Supplementary Fig. 2), indicating quantitative staining over a range of 0.40 – 7.0 pg DNA per nucleus. Only hen and trout standards (Figs. 1 A,B in main text) were used in the staining batch with *Ls* Gulen and Maine specimens. We note that we prefer the IOD measurements of the hen standard to compute pg of DNA per nucleus of *L. salmonis salmonis*, because the hen is regarded as the most reliable standard, even when a series of standards reveals quantitative uptake of the Schiff reagent (Rasch 1985, Wyngaard, pers. observation).

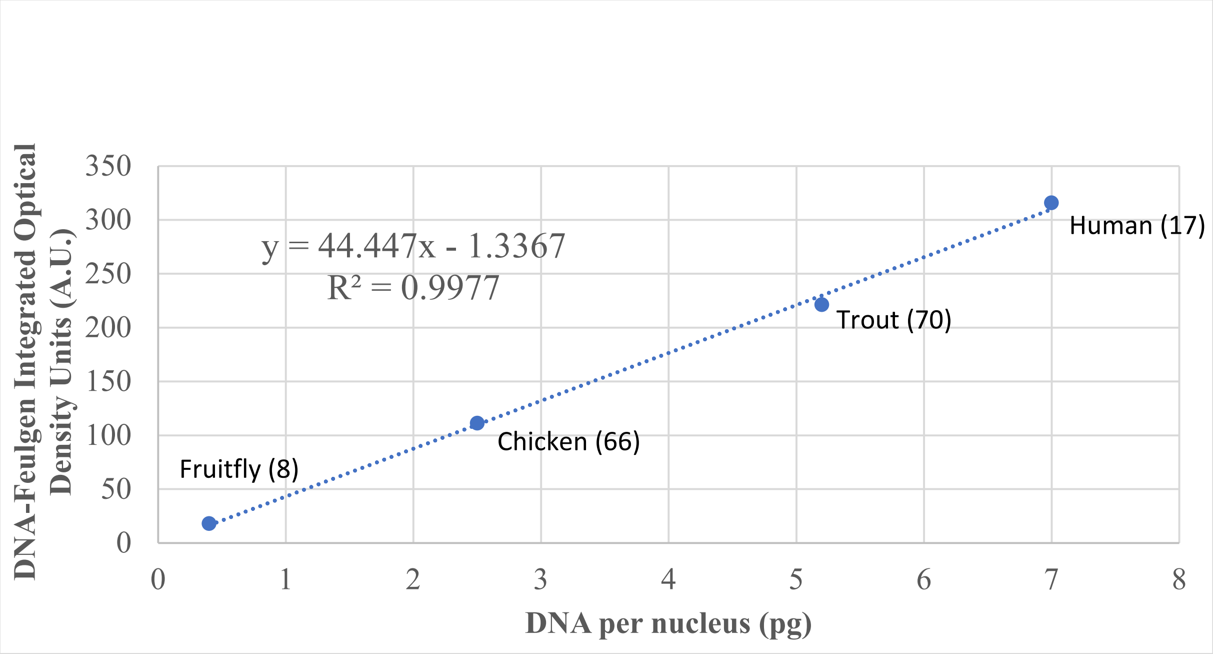

Supplementary Figure 2. Calibration of genome size estimates of standards used in DNA-Feulgen Image Analysis Densitometry. Values in picograms of standards are for the wild type fruitfly of female *Drosophila melanogaster* (Mulligan & Rasch, 1980), erthrocytes of chicken (*Gallus domesticus*) and trout rainbow trout (*Oncorhynchus mykiss*, previously named *Salmo gairdineri irideus* (Rasch, 1985) and mono-nucleated cells of male human *Homo sapiens* (Gregory, 2021). Values of arbitrary units (A.U.) of integrated absorbance at 560 nm, except for human, which was measured at 605 nm. Number of nuclei shown in parentheses. Coefficients of variation varied from 3.5 – 3.9%. References for genome sizes of standards are included because there are multiple references and genome sizes reported for each standard in Gregory (2021).

The Bioquant software used to measure IODs has a conservative estimate of resolution of 0.5 pg DNA per nucleus according to the manufacturer. Practical experience indicates the resolution to be closer to 0.25 pg DNA per nucleus (personal observation, GAW). The mean IOD value of the hen was used to convert the IODs of each *L. salmonis salmonis* specimen to picograms, using the following equation:

$$pg_c = (pg_s/IOD_s) \times IODc$$

where pg_c is the unknown amount of pg DNA per nucleus in *L. salmonis* pg_s , 2.5 pg is the amount of DNA in the standard hen nucleus, IOD_s is the average IOD value of the hen, and IODc is the IOD value of *L. salmonis*.

Photographic images. Photographs were taken at 100X magnification with a Nikon Eclipse Ti-2 compound microscope equipped with a PlanApo objective (N.A. 1.45) and QImaging DS RI2 camera and saved as .tiff files.

Statistical analyses

Differences in nuclear DNA content of somatic tissues of hybrid nauplii (FCM Run 1 and 2) and *Ls* Gulen germinal (eggs and sperm) or somatic tissues of hybrid and *Ls* Gulen adult males and females (FCM Run 3-4), as well as those of *Ls* Gulen adults obtained using FIAD, were analyzed by Students *t*-test. Analysis of variance (ANOVA) was used to detect significant differences in nuclear DNA content of somatic tissue of adult wild caught and laboratory hybrid strain *L. salmonis salmonis* (FCM Run 5) using fluorescence (FL) PI values as dependent variable and gender and strain (laboratory or wild) as factors. In Exploratory Data Analysis (EDA), Grubbs' test was used to detect presence of outliers and Levene's and Shapiro-Wilk tests were used to test homogeneity of variances among groups. Statistical analyses were performed using IBM SPSS Statistics v.25 software. Data are reported as mean ± standard error (SE). The Shapiro-Wilks test applied to nuclei within each of 10 specimens measured using FIAD revealed no departures from normality. Differences between male and female genome sizes based on FIAD were tested using two sample, two-tailed Student *t*-test.

References

Clower, M.K., Holub, A.S., Smith, R.T. & Wyngaard, G.A. Embryonic development and a quantitative model of programmed DNA elimination in Mesocyclops edax (S.A. Forbes, 1891) (Copepoda: Cyclopoida). *J. Crustac. Biol.* **36**, 661-674 (2016).

Doležel, J., Bartoš, J., Voglmayr, H. & Greilhuber, J. Nuclear DNA and genome size of trout and human. *Cytometry* **51**, 127–128 (2003).

Duerr, J.S. Antibody staining in *C. elegans* using "freeze-cracking". *J. Vis. Exp.* **80**, e50664; 10.3791/50664 (2013).

Gregory, T.R. Animal Genome Size Database. http://www.genomesize.com (2021).

Hamre, L.A., Glover, K.A. & Nilsen, F. Establishment and characterisation of salmon louse (Lepeophtheirus salmonis (Kroyer 1837)) laboratory strains. *Parasitol. Int.* **58**, 451-460 (2009).

Hardie, D.C., Gregory, T.R. & Hebert, P.D.N. From pixels to picograms: A beginner's guide to genome quantification by Feulgen image analysis densitometry. *J. Histochem. Cytochem.* **50**, 725-749 (2002).

Jeffery, N.W. Genome size diversity and evolution in the Crustacea. *Ph.D Thesis*, University of Guelph; 257 pp. https://atrium.lib.uoguelph.ca/xmlui/handle/10214/9216 (2015).

Mulligan, P.K. & Rasch, E.M. The determination of genome size in male and female germ cells of *Drosophila melanogaster* by DNA-Feulgen cytophotometry. *Histochemistry* **66**, 11–18 (1980).

Rasch, E.M. DNA "standards" and the range of accurate DNA estimates by Feulgen absorption microspectrophotometry in *Advances in Microscopy* (eds. Cowden, R.R, and Harrison, F.W.) 127-166 (Alan R. Liss, Inc.1985).

Rasch, E.M. & Wyngaard, G.A. Genome sizes of cyclopoid copepods (Crustacea): evidence of evolutionary constraint. *Biol. J. Linn. Soc.* **87**, 625-635 (2006).

Tiersch, T.R., Chandler, R.W., Kallman, K. & Wachtel, S.S. Estimation of nuclear DNA content by flow cytometry in fishes of the genus Xiphophorus. *Comp. Biochem. Physiol. B* **94**, 465-468 (1989).

Vindeløv, L.L., Christensen I.J. & Nissen, N.I. A detergent-trypsin method for the preparation of nuclei for flow cytometric DNA analysis. *Cytometry* **3**, 323-327 (1983).
